# The Value of the Near‐Infrared Fluorescent Probe ErNP@SiO2‐ICG in the Diagnosis of Malignant Pleural Effusion

**DOI:** 10.1155/carj/2562288

**Published:** 2026-02-17

**Authors:** Chuchu Xu, Xiaoxia Wang, Xingya Yan, Xiaona Yin, Xi Wang, Fangbin Du, Yinling Jiang, Xiaoqiong Wang, Yongsheng Wang

**Affiliations:** ^1^ Department of Pulmonary and Critical Care Medicine, The Second People’s Hospital of Hefei, Hefei Hospital Affiliated to Anhui Medical University, Hefei, 230011, Anhui, China, ahmu.edu.cn; ^2^ Department of Respiratory and Critical Care Medicine, The Affiliated Hefei Hospital of Anhui Medical University (Hefei no. 2 People’s Hospital), Hefei, 230011, Anhui, China; ^3^ Department of Respiratory and Critical Care Medicine, The Fifth Clinical Medical College of Anhui Medical University, Hefei, 230032, Anhui, China

**Keywords:** ErNP@SiO2-ICG, indocyanine green, malignant pleural effusion, near-infrared fluorescence, rare earth nano

## Abstract

**Objectives:**

Malignant pleural effusions (MPEs) present a significant clinical challenge and are associated with a poor prognosis, frequently observed in patients with advanced malignancies. Conventional diagnostic techniques for identifying MPEs exhibit limitations in both accuracy and sensitivity. To differentiate between benign and MPEs, the clinical applicability of the ErNP@SiO2‐ICG rare earth nano–nano near‐infrared (NIR) fluorescence probe was investigated.

**Methods:**

Through solution chemistry processes, vacuum treatment with heat, and the synthesis of a core–shell design, an exceptional rare earth nano NIR fluorescent probe was developed in this study, which showed the capacity to target tumors precisely. After that, a prospective research was conducted with a cohort of 90 patients; 20 of them were excluded because of unclear diagnoses. Every participant had a thoracoscopic biopsy for histological analysis, a cytological assessment of pleural effusions, and an evaluation of the recently developed dyes. R programming, GraphPad Prism, and Microsoft Excel were used when carrying out statistical inspection of the obtained data.

**Results:**

ErNP@SiO2‐ICG particles experienced a size of 176.1 ± 0.2 nm. Their emission peak was located at 550 nm in relation to their fluorescence spectra, and their scanning electron microscopy image demonstrated uniform particle size and distribution with NIR fluorescence characteristics. The optimum time and concentration for color development were 2 μL and 1 h, respectively. The fluorescence imaging and cytological investigation of pleural effusions differed significantly (*p* < 0.001) in 35 scenarios of MPEs, 35 cases of benign pleural effusions, and 20 cases of unexplained pleural effusions among the 90 participants. The area under the ROC curve for fluorescence imaging of ErNP@SiO2‐ICG was 0.814 (95% confidence interval: 0.708–0.920). The fluorescence imaging sensitivity and specificity of ErNP@SiO_2_‐ICG were 0.814 (95% CI: 0.652–0.872), while the area under the ROC curve for pleural fluid cytology was 0.729 (95% CI: 0.607–0.850). The area under the ROC curve for the ErNP@SiO_2_‐ICG NIR fluorescent probe showed good compliance with pathologic findings (Kappa = 0.629, *p* < 0.001). The results of the confusion matrix constructed based on this threshold showed a positive predictive value of 82.40% and a negative predictive value of 80.60%, with a false‐positive rate of 17.60% and a false‐negative rate of 19.40%.

**Conclusion:**

The ErNP@SiO2‐ICG rare earth‐doped nano‐probe for NIR fluorescence imaging exhibits exceptional accuracy in the detection of MPE, thereby providing an innovative technological approach for the future identification of this condition.

## 1. Introduction

Pleural effusion (PE) is a prevalent clinical manifestation associated with a range of medical conditions, such as pneumonia, neoplastic diseases, tuberculous pleuritis, systemic illnesses, and traumatic events. This phenomenon arises from an abnormal accumulation of fluid within the pleural space [1, 2]. Malignant pleural effusion (MPE) is primarily attributed to malignant tumors that either originate in or metastasize to the pleura [3]. Among these, lung cancer is the predominant type, accounting for approximately one‐third of all cases [4, 5], with a global incidence estimated at 70 cases per million individuals [6]. Despite the significant economic burden posed by high rates of hospital readmissions and extensive resource utilization, the healthcare system must remain vigilant regarding the implications of MPEs [7]. The classification of PE as either benign or malignant plays a crucial role in determining the prognosis for patients. Consequently, precise differentiation between these two types of PEs is essential [8, 9]. From the date of MPE diagnosis, the median survival period for cancer patients is 3–12 months. Typically, MPE patients progress to stage IV, with a poor prognosis and low quality of life [10, 11]. Pleural biopsy and cytology are the current gold standard for the diagnosis of MPE [12]. However, the sensitivity of cytological assessment is approximately 60%, with diagnostic accuracy being contingent on the pathologist’s expertise and influenced by variables, such as the nature of the primary tumor, the volume of the sample, and the techniques employed in processing [13, 14]. A variety of tumor biomarkers, including CEA, CA125, CA15‐3, and CA19‐9, can be utilized for the diagnosis of MPE; however, these biomarkers demonstrate inadequate organ specificity and sensitivity in identifying MPE [15]. The utilization of near‐infrared (NIR) fluorescence imaging has attracted considerable attention owing to its various benefits, such as extended excitation wavelengths, enhanced tissue penetration depth, and improved contrast in biological environments. This positions NIR fluorescence imaging as a promising modality for the early surveillance and identification of tumors [16–18]. As the only NIR photothermal reagent approved by the US Food and Drug Administration (FDA) for clinical research, Index Jingjing Green (ICG) has been widely studied [19] in the field of photothermal therapy (PTT) [20] anticancer due to its excellent tissue permeability, biological safety, and photothermal conversion efficiency [21, 22]. Nonetheless, ICG encounters several obstacles in clinical utilization, including accelerated in vivo metabolism, insufficient stability, and a deficit in targeted delivery [23, 24]. Nanoparticles are increasingly recognized as optimal candidates for diverse cellular imaging techniques due to their intrinsic optical characteristics [25]. Lanthanides exhibit prolonged luminescence lifetimes and unique 4f‐4f electronic transitions, resulting in emission spectra characterized by narrow and defined bands, making them widely utilized in the development of fluorescence‐based applications [26]. Research indicates that nanoparticles doped with rare earth ions exhibit remarkable upconversion characteristics, and their biocompatibility can be enhanced through a silica coating, with dimensions varying from several tens to several hundreds of nanometers [27–30]. In our experimental framework, we incorporated rare earth‐doped nanomaterials utilizing SiO2 as the nanocarrier to encapsulate ICG dye, thereby safeguarding its fluorescent characteristics and improving photostability. The dimensions and fluorescence emission spectrum of ErNP@SiO2‐ICG were evaluated through material characterization techniques. Subsequently, we assessed the tumor‐targeting efficacy of ErNP@SiO2‐ICG, identifying its optimal concentration and duration of exposure. Finally, we conducted clinical validation by selecting samples from 90 patients scheduled for thoracoscopic surgery to evaluate the diagnostic performance of the novel approach.

## 2. Methods

### 2.1. Indocyanine Green Dye and Pleural Fluid Samples

Xi’an Kaixin Biotechnology Company obtained samples of pleural fluid and used them to extract indocyanine green (ICG) dye. Additionally, the company developed the ErNP@SiO2‐ICG synthesis method. Both samples were kept at −20°C before use. The Department of Respiratory and Critical Care Medicine at the Second People’s Hospital of Hefei City provided the pleural fluid samples. The hospital used each specimen for an internal medicine thoracoscopy and pathology diagnosis. The pathology reports were then issued by the same hospital. Inclusion criteria were as follows: (1) patients over 18 years of age with chest CT or thoracic ultrasound revealing PE; (2) all patients must have undergone standard preoperative evaluations, including complete blood count, biochemical tests, coagulation profile, and electrocardiography; and (3) absence of any contraindications for thoracoscopic surgery related to internal medical conditions. Exclusion criteria were as follows: (1) patients who cannot withstand surgical intervention; (2) patients with transudative PE due to conditions, such as hypoalbuminemia or congestive heart failure; (3) patients with a diagnosed underlying condition necessitating endoscopic intervention; and (4) patients with significant cardiovascular disease, severe hepatic or renal impairment, or psychiatric disorders. Thoracoscopic biopsy procedures are conducted by the chief physician of our department. All participants undergo biopsy performed by the same physician, and two designated pathologists are tasked with analyzing and interpreting the specimens, which includes microscopic examination of tissue samples and other pathological assessments for disease diagnosis.

### 2.2. ErNP@SiO2‐ICG Synthesis and Personality Development

**FIGURE 1:**
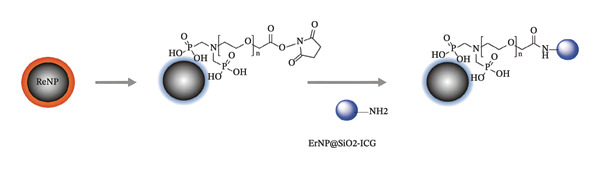
A schematic diagram of the ErNP@SiO2‐ICG synthesis.

Xi’an Kaisheng Company supplied ICG NIR fluorescent dye, and its Nanosynthesis Laboratory synthesized ErNP@SiO2‐ICG. This process involved the formulation of a Na and F solution, followed by vacuum heating and the integration of Ln, Na, and F to synthesize NaYF4, Yb, and Er nanoparticles. (1) Synthesis of ErNPs—Preparation of the reaction solution: Introduce the ErNP solution into a 100‐mL three‐neck flask. Dissolve 0.148 g of NH_4_F in 10 mL of methanol while stirring until fully dissolved. Weigh 0.1 g of NaOH, dissolve it in 2.5 mL of methanol, and utilize an ultrasonic cleaner for extended sonication until the solution is completely homogeneous. Combining the Ln and NaF solutions: Add the Ln(oleate)3 solution to a 100‐mL three‐neck flask equipped with a high‐temperature magnetic stirrer, thermowell, and gas inlet tube, ensuring gentle stirring. Incorporate the dissolved NaOH solution into the NH_4_F mixture and mix thoroughly via vortexing for 10 s. Promptly transfer the combined solution to the three‐neck flask. Secure the flask in an electric heating mantle, insert a temperature probe, connect the argon gas flow line, and adjust the flow rate accordingly. (2) SiO2 Shell Coating—Hydrolysis reaction: Gradually introduce 10 mL of anhydrous ethanol and 2 mL of TEOS into the ErNP‐containing solution to initiate hydrolysis, adding NH_4_OH as a catalyst to expedite the hydrolysis of TEOS. Reaction conditions: Maintain the reaction temperature at 50°C with stirring for 30 min. Subsequently, increase the temperature to 80°C and continue stirring for 1 h to achieve a consistent SiO2 shell formation. (3) ICG Loading—Dissolve ICG in methanol to create a solution of the desired concentration: Incorporate ErNP@SiO2 into the ICG solution and stir gently to ensure thorough mixing. Employ a vacuum pump to evaporate the solvent, promoting an even distribution of ICG within the SiO2 shell. (4) Post‐treatment and Characterization—Centrifugation and washing: Transfer the resultant mixture to a 50‐mL centrifuge tube and centrifuge at 5000 rpm for 10 min. Discard the supernatant, then add 50 mL of ethanol for washing, followed by another centrifugation to eliminate any unbound ICG. Following the application of an amino‐modified silylating reagent to the nanoparticles, the reaction was stirred with ICG for 24 h at room temperature to purify the particles. The particles were then dispersed in PBS for further use, and the synthesis process is illustrated in Figure 1. The ErNP@SiO2‐ICG was stored in a refrigerator at minus 20°C and protected from light.

### 2.3. Cell Culture and Pregnancy

A549 human non–small cell lung carcinoma cells (CL‐0016), along with the H1299 and H1975 non–small cell lung carcinoma cell lines, as well as human type II alveolar epithelial cells (CP‐H209), were acquired from Wuhan Punoise Biotechnology Co. The type II alveolar epithelial cells were maintained in RPMI‐1640 medium supplemented with 10% fetal bovine serum and 1% penicillin–streptomycin. In contrast, the A549, H1299, and H1975 cell lines were cultured in DMEM enriched with high glucose and 10% fetal bovine serum. Both cell types were sustained in complete medium containing 10% fetal bovine serum and 1% penicillin–streptomycin, with the culture medium replaced every two to 3 days based on the cells’ developmental stage. Upon reaching 80%–90% confluence on the culture plates, a cell passage was performed. Following digestion with 0.25% trypsin, the cells, which had attained 80%–90% confluence on the culture surface, were passaged at a 1:3 dilution ratio. Their uniform distribution was confirmed microscopically, and they were subsequently returned to a 5% CO2 atmosphere at 37°C for continued growth.

### 2.4. ErNP@SiO2‐ICG Optimal Concentration and Time in Vitro

To ascertain the most effective concentration and duration of action in vitro, we utilized A549 (CL‐0016), H1299, and H1975 cell lines, which were cultured in a 24‐well plate and maintained in a cell culture incubator at 37°C with 5% CO2. The culture medium consisted of DMEM with high glucose, supplemented with 10% fetal bovine serum and refreshed with 1% penicillin and streptomycin solution (1 mL) every two days. Fluorescence staining was performed when the cell density approached 80%. The ErNP@SiO2‐ICG was reconstituted at ambient temperature and prepared into five concentration gradients of 0.5, 1.0, 2.0, 3.0, and 5.0 μL to identify the optimal fluorescence labeling concentration for clinical reference. Following this, we established identical experimental conditions across five time intervals: 10, 30 min, 1, 2, and 24 h. The optimal concentration of the fluorescent dye was then administered to each well, and the ideal exposure duration was evaluated.

The specific staining steps were as follows: The cell density was about 80%, and the dye was co‐incubated with the cells according to the specific time and concentration mentioned above. After the incubation was completed, the culture mixture was discarded, the slides were washed with PBS, fixed with 4% paraformaldehyde for 30 min, PBS was cleared twice, and 1 mL of PBS was added to each well to keep the cells in a wet state, waiting for uniform imaging. Use DMEM high‐glucose medium for three groups and RPMI‐1640 complete medium for the other group. Change the liquid every 2‐3 days until the cells have reached about 80% confluence. Follow the same procedure as outlined above for staining, fixation, wetting with PBS, and waiting for imaging confirmation to determine whether the synthetic Metro dyes effectively target tumors.

### 2.5. Evaluation of ErNP@SiO2‐ICG in Malignant Pleural Effusion

**FIGURE 2:**
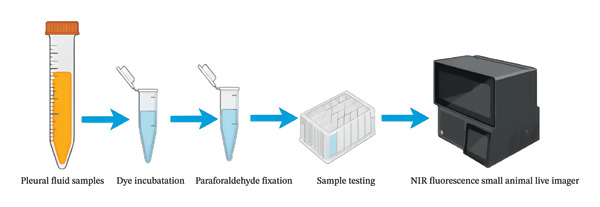
Diagram of the experimental process.

On the day of the thoracoscopic procedure, approximately 20 mL of PE should be collected. Subject the fluid to centrifugation at 1200 rpm for 15 min. Discard the supernatant while retaining the cellular component at the bottom. Resuspend the pellet in 2 mL of phosphate‐buffered saline (PBS). Transfer the cell suspension to a centrifuge tube and incorporate 2 μL of ErNP@SiO2‐ICG NIR fluorescent dye. Incubate the mixture in a cell culture incubator set to 5% CO2 and 37°C for 1 h to ensure cellular viability. After incubation, centrifuge the cells, discard the supernatant, and preserve the cell pellet. Wash the cells with PBS, centrifuge again, and remove the supernatant. Fix the cells in 4% paraformaldehyde for 30 min. Following centrifugation, discard the supernatant and store each centrifuge tube containing 1 mL of PBS in a refrigerator at 4°C. Proceed to obtain uniform imaging (Figure 2).

### 2.6. Statistical Analysis

**FIGURE 3:**
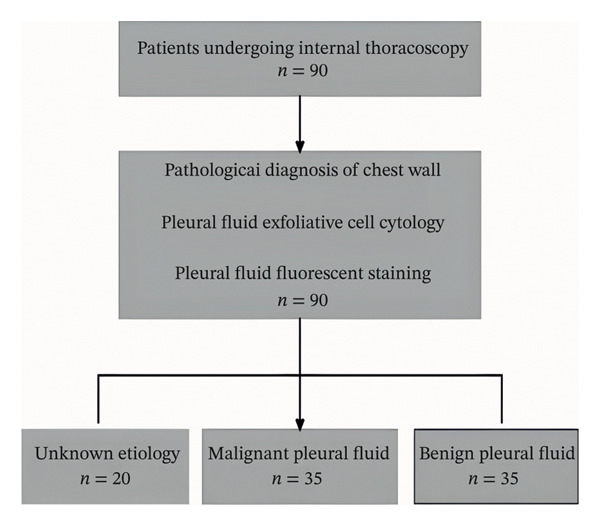
Pleural fluid sample selection process.

The study was carried out with authorization from the Ethics Committee of the Second People’s Hospital of Hefei City, identified by the ethical number 2023—Scientific Research—048. Informed consent was obtained from all patients, who were admitted to the Department of Respiratory and Critical Care Medicine at the same facility. Additionally, they provided consent specifically for thoracoscopic procedures. Each participant underwent pleural fluid exfoliative cytology analysis and wall pleural biopsy. A total of 90 patients were recruited, all of whom satisfied the criteria for thoracoscopic intervention. Based on the thoracoscopic pathology findings, 20 cases were classified as having an unknown etiology, 35 cases exhibited MPE, and 35 cases had benign PE. All pleural fluid specimens were analyzed for exfoliative cytology at Hefei No. 2 Hospital, and the same specimens were processed with ErNP@SiO2‐ICG for further examination at the Suzhou Nano Research Institute of the Chinese Academy of Sciences on a biochemical platform (Figure 3). Data collection was performed using Microsoft Excel, encompassing baseline characteristics of the participants, as well as fluorescence intensity and absorbance measurements of ErNP@SiO2‐ICG, alongside the results from pleural fluid exfoliative cytology. Statistical analysis was conducted with R Version 4.3.1. Data conforming to a normal distribution were presented as mean and standard deviation and compared between groups using *t*‐tests. Non‐normally distributed quantitative data were reported as median P50 (P25 and P75) and analyzed with the Mann–Whitney *U*‐test. Categorical data were expressed as percentages, with intergroup comparisons conducted via chi‐square tests. Graphical representation of the results was achieved using GraphPad Prism 9 and R Version 4.3.1. A *p* value of less than 0.05 was deemed statistically significant for all analyses.

## 3. Results

### 3.1. ErNP@SiO2‐ICG Formation and Characterization

The 200 kV projection electron microscopy analysis revealed that the size of the ErNP@SiO2‐ICG particles matched the dynamic light scattering test result of 176.18 ± 0.2 nm. Additionally, the dynamic particle size scanning indicated that the inclusion of ICG did not have a significant impact on the nanoparticle size. The diameters of the ErNP@SiO2 particles and the ErNP@SiO2‐ICG particles were measured at 152.0 ± 0.1 nm and 176.1 ± 0.2 nm, respectively, showing that they were similar in size. The fluorescence spectra of ErNP@SiO2‐ICG displayed an emission peak at 550 nm, while the scanning electron microscope image revealed uniform particle size and consistent distribution, meeting the experimental requirements (Figure 4).

FIGURE 4(a) The hydrated particle size of ErNP@SiO2 is 152.0 ± 0.1 nm. (b) The hydrated particle size of ErNP@SiO2‐ICG is 176.1 ± 0.2 nm. (c) The ErNP@ SiO2 fluorescence spectrum revealed an emission peak located at 550 nm. (d) Electron micrographs of the ErNP@SiO2 particles.

**Figure:**
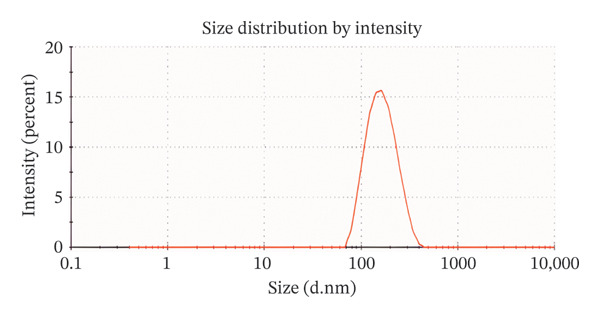
(a)

**Figure:**
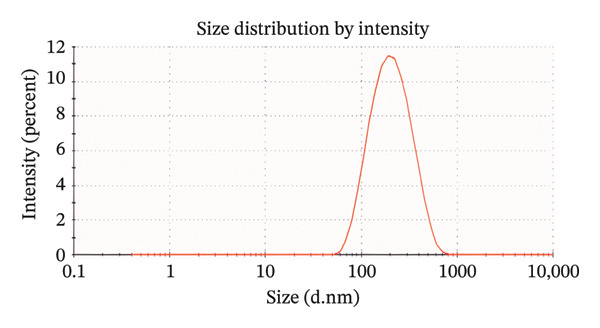
(b)

**Figure:**
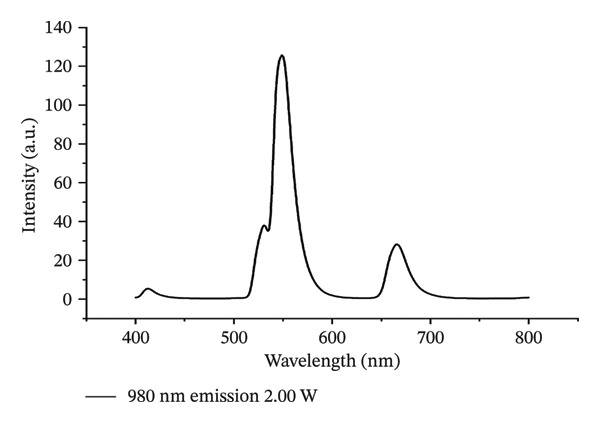
(c)

**Figure:**
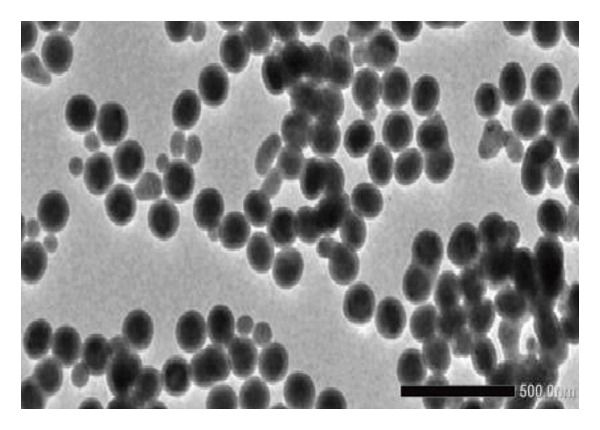
(d)

### 3.2. Mapping the Optimal Concentration and Time of ErNP@SiO2‐ICG

**FIGURE 6:**
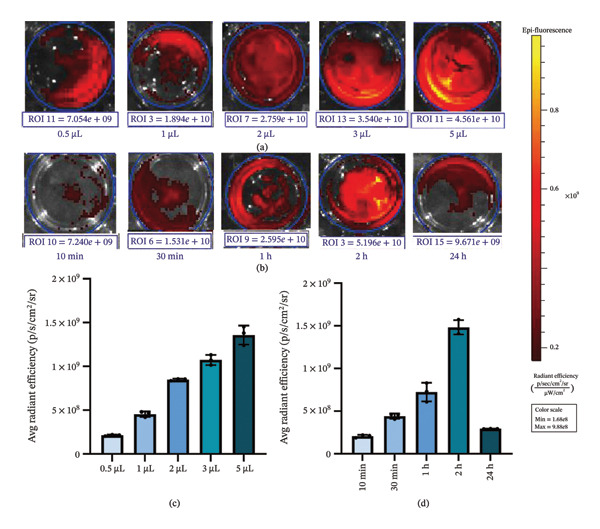
(a, b) Mean radiation efficiency of malignant hydrothorax treated with ErNP@ SiO2‐ICG at different concentrations (0.5, 1, 2, 3, and 5) and at different time points (10 min, 30, 1, 2, and 24 h). (c) The average radiation efficiency fluorescence values of malignant pleural effusion cells at different concentrations. (d) The average radiation efficiency fluorescence values of malignant pleural aqueous cells at different time points.

In this study, we utilized three human non–small cell lung carcinoma cell lines—A549, H1299, and H1975—as experimental models, with human type II alveolar epithelial cells (ATII) serving as the control. Employing in vivo small animal imaging with NIR fluorescence, we detected notable variations in the uptake of the ErNP@SiO_2_‐ICG probe among the different cell types. At a concentration of 1 × 10^6^ cells per well and following a one‐hour incubation period, the human type II alveolar epithelial cells exhibited no uptake of ErNP@SiO_2_‐ICG. In contrast, the human malignant lung cancer cell lines A549, H1299, and H1975 demonstrated significant uptake of the ErNP@SiO_2_‐ICG probe under identical conditions (Figure 5(a)). Moreover, the malignant cells showed elevated average radiative efficiency compared to the benign cells, with distinct average radiative values noted across the various tumor cell types (Figure 5(b)). This suggests that the ErNP@SiO_2_‐ICG probe possesses promising tumor‐targeting capabilities. We used A549 human non–small cell lung cancer cells as our cell model. To determine the optimal reaction concentration of ErNP@SiO2‐ICG, we tested five different concentrations: 0.5, 1, 2, 3, and 5 μL. We found that 2 μl was the best concentration for color development. At 3 μL, we observed a fluorescence exposure phenomenon, confirming that 2 μL was indeed the optimal concentration for color development (Figure 6(a)). Figure 5(b) illustrates the impact of color development when 2  μl of fluorescent dye is added per well at different time intervals (10, 30 min, 1, 2, and 24 h). It is evident that the most effective duration for the fluorescent dye to act is approximately 1 h (Figure 6(b)), leading to the most favorable color development outcome. Over time, fluorescence attenuation is observed. This phenomenon occurs due to the determination that the optimal concentration and time for color development are 2 μL and 1 h, respectively. Figure 6(c) displays the average radiation efficiency fluorescence values of malignant pleural fluid cells at different concentrations, while Figure 6(d) shows the average radiation efficiency fluorescence values at different time points.

FIGURE 5(a) Normal human cells (ATII) showed no uptake of ErNP@SiO2‐ICG after incubation. Human malignant cell lines (A549, H1299, and H1975) showed significant uptake of ErNP@SiO2‐ICG under similar staining and imaging; (b) malignant cells demonstrated higher average radiative efficiency values than benign cells, with distinct average radiative values across different tumor cell types.

**Figure:**
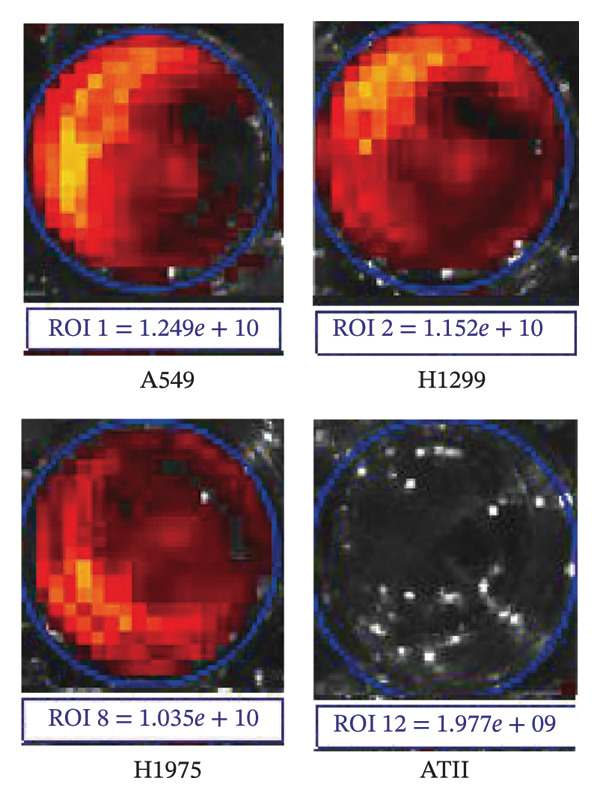
(a)

**Figure:**
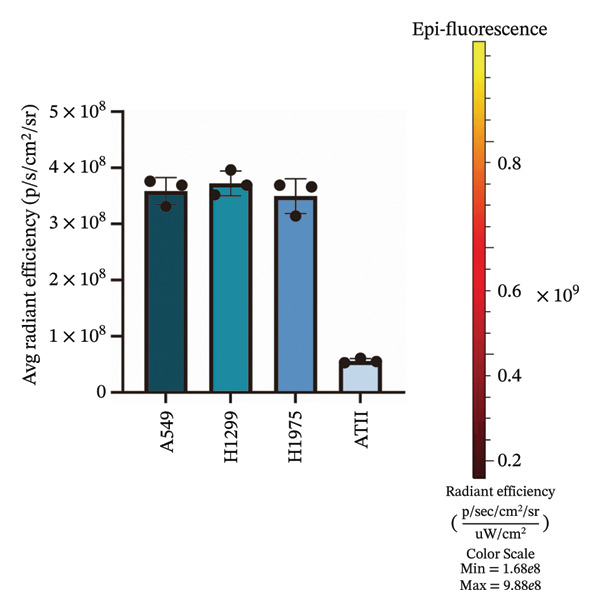
(b)

### 3.3. The Ability of the New ErNP@SiO2‐ICG Staining Method in the Diagnosis of Malignant Pleural Effusion

A cohort of 90 patients presenting with idiopathic PE was enrolled in this investigation. The participants underwent staining utilizing the novel ErNP@SiO2‐ICG technique, while PE samples were dispatched to our pathology department for cytological evaluation. The definitive diagnostic criterion was the histopathological report of the parietal pleura obtained through thoracoscopic intervention. These reports were classified as indicating MPEs, benign PEs, or indeterminate diagnoses. Statistical analysis revealed 35 instances of MPEs, 35 instances of benign PEs, and 20 instances of undetermined PEs among the 90 participants. Within the 35 cases of MPE, 26 were identified as adenocarcinomas, 6 as squamous cell carcinomas, 1 as a malignant neoplasm of epithelial origin, and 2 as pleural mesotheliomas. Among the 35 benign PEs, 22 were attributed to tuberculous pleuritis, and 13 were due to inflammatory pleuritis and pyothorax. To evaluate the diagnostic efficacy of the dye for MPEs, baseline data were compiled for 70 patients, encompassing age, sex, and the presence of chronic comorbidities, such as hypertension, diabetes, cardiac disorders, and cerebral infarction, alongside the findings from PE cytology and fluorescence imaging (refer to Table 1). There were no significant statistical differences between the two cohorts regarding age, gender, and chronic underlying conditions (*p* > 0.05). Conversely, fluorescence imaging and pleural fluid cytology exhibited notable disparities (*p* < 0.001). We conducted a ROC analysis for both fluorescence imaging and pleural fluid cytology utilizing R software (Figure 7). The sensitivity and specificity for fluorescence imaging were recorded at 80.00% and 82.90%, respectively, with an area under the ROC curve of 0.814 (95% CI: 0.708–0.920). In contrast, the area under the ROC curve for pleural fluid cytology was determined to be 0.729 (95% CI: 0.607–0.850) (Table 2). The ErNP@SiO2‐ICG NIR fluorescent probe demonstrated a strong correlation with pathological findings (Kappa = 0.629, *p* < 0.001). The confusion matrix generated at this threshold revealed a positive predictive value of 82.40%; the negative predictive value was 80.60%, with a false‐positive rate of 17.60% (indicating the percentage of benign cases misclassified as malignant) and a false‐negative rate of 19.40% (indicating the percentage of malignant cases misclassified as benign).

**TABLE 1:** Comparison table of baseline and observational indicators for dual patient groups.

Variant	BPE (*n* = 35)	MPE (*n* = 35)	*χ* ^2^/Z	*p*
Age (years)	61.06 ± 15.52	68.80 ± 14.89	2.218	0.493

Sex			1.556	0.212
Male	25 (71.4)	20 (57.1)		
Female	10 (28.6)	15 (42.9)		

Chronic disease history			3.684	0.055
Yes	12 (34.3)	20 (57.1)		
No	23 (65.7)	15 (42.9)		

Pleural fluid cytological			20.741	< 0.001
Positive	0 (0)	16 (45.7)		
Negative	35 (100)	19 (54.3)		

Fluorescence imaging			27.680	< 0.001
Positive	6 (17.1)	28 (80)		
Negative	29 (82.9)	7 (20)	2.93	
BMI(kg/m^2^)	21.51 ± 2.93	22.57 ± 3.04	1.491	0.70

*Note:* The chronic history mainly includes hypertension, cerebral infarction, coronary heart disease, diabetes mellitus, and other chronic diseases. Thoracic Cytology: Conventional cytology and diagnosis of pleural effusion; fluorescence imaging: fluorescence absorption of ErNP@ SiO2‐ICG by malignant cells.

Abbreviations: BPE, benign pleural effusion group; MPE, malignant pleural effusion group.

FIGURE 7(a) A confusion matrix for predicting benign or malignant appearance of pleural effusion by fluorescence imaging as an indicator. (b) The receiver operating characteristic curve of the ERNP@SiO2‐ICG NIR fluorescence in a malignant pleural effusion.

**Figure:**
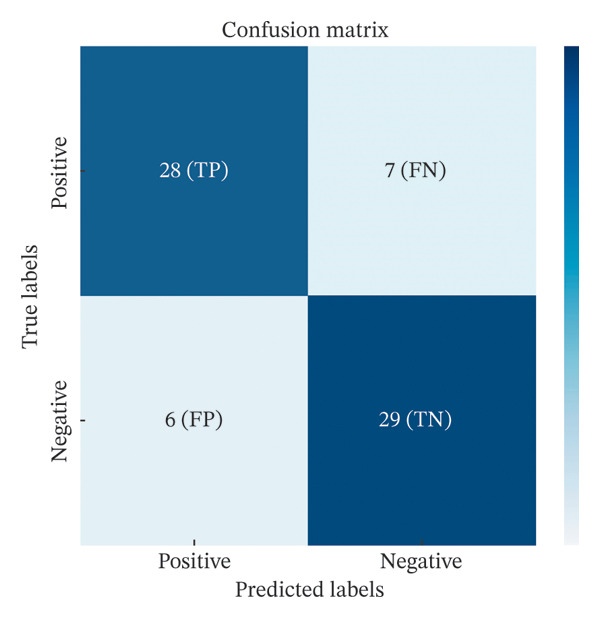
(a)

**Figure:**
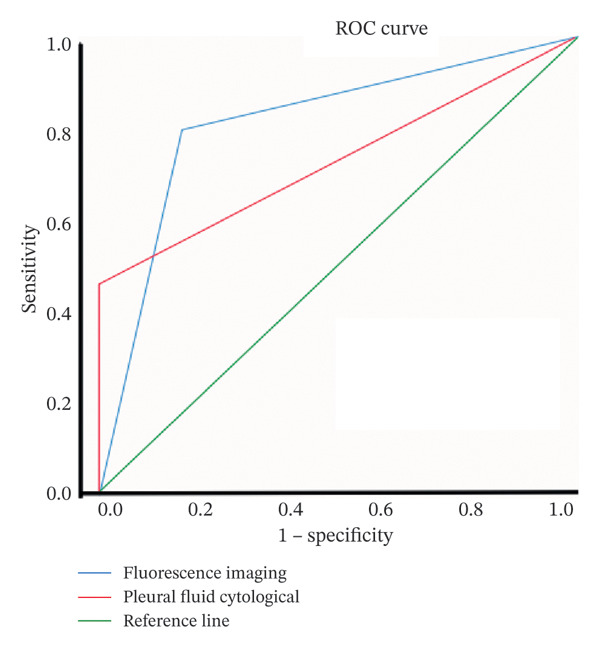
(b)

**TABLE 2:** Analysis of the prediction effect of different indicators.

Diagnostic method	Sensitivity (%)	Specificity (%)	Positive predictive value (%)	Negative predictive value (%)	AUC	*p*	95% CI
Pleural fluid cytological	45.70	100	100	64.80	0.729	0.001	0.607–0.850
Fluorescence imaging	80.00	82.90	82.40	80.60	0.814	< 0.001	0.708–0.920

*Note:* Thoracic cytology involves conventional cytology for the diagnosis of pleural effusion. In addition, fluorescence imaging is used to detect the fluorescence absorption of ErNP@SiO2‐ICG by malignant cells.

## 4. Discussion

In the case of PE, particularly when the cellular yield is minimal, the cytologic characteristics may closely resemble those of mesothelial cells, complicating the identification process. Furthermore, the intricacies involved in cytological assessment, coupled with the subjective interpretation by pathologists, render the diagnostic procedure protracted and vulnerable to personal biases, which can ultimately skew the outcomes [31]. In this investigation, the area under the ROC curve for conventional cytological analysis of pleural fluid in diagnosing MPE was found to be 72.90%, with a sensitivity of 45.70%, indicating a suboptimal diagnostic performance. Earlier, a research team led by Xiaoqiong Wang developed a NIR fluorescent probe, IR808‐MnO, by integrating NIR fluorescence tumor‐targeting technology with mesoporous manganese oxide, achieving a diagnostic accuracy of 75.7% for MPE [32]. The instability of fluorescence and vulnerability to interference in this design, particularly in patients with pyothorax characterized by an abundance of necrotic cellular material, coupled with the elevated production costs associated with IR808, have impeded its broader application. In contrast, Cy7 (Cyanine 7) is a prominent fluorescent dye extensively utilized in the fields of biomedical research and bioimaging. It exhibits a high quantum yield of fluorescence and remarkable photostability, enabling it to retain fluorescence intensity over extended periods of exposure. The emission wavelength of Cy7 is situated in the NIR spectrum (approximately 775 nm), which effectively reduces background noise in biological imaging, thus rendering it especially beneficial for imaging at greater tissue depths. Nonetheless, the synthesis of Cy7 can be relatively intricate and expensive, which may restrict its widespread utilization in certain applications. Under specific circumstances, Cy7 is susceptible to quenching phenomena, leading to signal degradation, particularly in environments with high concentrations. Furthermore, certain derivatives of Cy7 may interact with nontarget molecules, resulting in nonspecific signals that can undermine experimental precision. Some Cy7 derivatives also demonstrate inadequate in vivo stability, being vulnerable to biological degradation, which constrains the temporal windows for imaging and detection. The primary benefit of ErNP@SiO_2_‐ICG resides in its core–shell nanostructure design. The SiO2 shell mitigates the intrinsic challenges associated with ICG’s poor stability and quenching susceptibility while serving as a functional integration platform. The Er^3+^‐doped core introduces distinctive upconversion luminescence properties. This architectural design not only enhances photostability, brightness, and efficacy but also provides multimodal imaging capabilities, improved biocompatibility, and significant potential for multifunctional integration.

This research indicates that the diagnostic efficacy of ErNP@SiO2‐ICG fluorescence staining for MPE achieved an impressive 81.4%. This notable enhancement is largely due to the shortcomings of traditional fluorescent probes, including organic dyes, which suffer from inadequate stability, low fluorescence intensity, and suboptimal biocompatibility, thereby limiting their applicability in clinical settings. The unique optical characteristics of rare earth elements, stemming from their f‐orbital electronic configurations, encompass distinct fluorescence emission peaks, prolonged fluorescence lifetimes, substantial Stokes shifts, and high resistance to photobleaching. These properties render rare earth elements a promising alternative to conventional fluorescent probes in clinical diagnostics. Furthermore, rare earth elements are recognized as exceptional bioimaging agents, exhibiting strong tissue penetration, minimal autofluorescence interference, and favorable biocompatibility. Certain rare earth ions also demonstrate significant fluorescence in the NIR spectrum, positioning them as optimal candidates for NIR imaging applications. The generation of NIR fluorescence, particularly through the process of upconversion, is facilitated by multiphoton absorption mechanisms. In a composite system comprising Yb^3+ and Er^3+, Yb^3+ initially absorbs a photon, subsequently transferring energy nonradiatively to Er^3+. This energy transfer enables Er^3+ to absorb a second photon, elevating it to a higher excited state. Upon transitioning back to a lower energy state, Er^3+ emits fluorescence within the NIR spectrum. A significant advantage of this upconversion process is the utilization of longer excitation wavelengths (e.g., 980 nm), which enhance tissue penetration while minimizing damage to biological tissues. Moreover, the shorter wavelength of fluorescence emitted by the upconversion material allows for improved separation of the fluorescent signal from the background, thereby enhancing imaging contrast and sensitivity. ICG has garnered considerable interest due to its excellent tissue permeability, biocompatibility, and photothermal conversion efficiency. Additionally, silicon dioxide nanomaterials demonstrate favorable biocompatibility, and their integration with ICG markedly enhances stability compared to IR808‐MnO, thereby improving the fluorescent staining efficacy of ErNP@SiO2‐ICG in the diagnosis of MPE. This research improved the stability, intensity, and biocompatibility of fluorescence by integrating rare earth elements Er^3+ and SiO2 with ICG fluorescent dye. Statistical evaluations indicated that this combination demonstrated exceptional diagnostic performance in identifying MPE, exhibiting enhanced sensitivity, specificity, positive and negative predictive values, as well as favorable ROC curves, all at a reduced cost. In contrast to conventional qualitative cytological examination of malignant cells found in pleural and abdominal fluids, the proposed method is more intricate and labor‐intensive. The rapid and efficient diagnostic approach introduced in this study, which involves only centrifugation, incubation, and washing, showed high precision and sensitivity toward malignant cells in pleural and abdominal effusions, significantly minimizing time expenditure. Consequently, ErNP@SiO2‐ICG emerges as an innovative fluorescent probe, serving as a sensitive, user‐friendly, cost‐effective, and time‐efficient tool for clinical detection, with extensive potential applications. However, the limitations of a small sample size and a single‐center design are acknowledged. Future research will include a multicenter study with an expanded sample size.

## 5. Conclusions

Although MPE is a complex pathology, cytological examination and serum tumor marker analysis are frequently employed in clinical practice to evaluate the nature of PEs as either benign or malignant. Nonetheless, even in cases of positive results, these methods often lack sufficient oncological insights to guide subsequent therapeutic strategies, primarily due to the inadequate sensitivity of cytological evaluations of pleural fluid. To address this limitation, the study integrates silica (SiO2) biocarriers with erbium (Er^3+)‐doped rare earth nanomaterials, resulting in the creation of ErNP@SiO2‐ICG rare copper nano‐nanoparticles. These NIR fluorescence probes exhibit remarkable photostability and solubility in aqueous environments, facilitating the high‐precision imaging of malignant pleural infections. This advancement paves the way for enhanced detection of MPEs in future applications.

## Author Contributions

Chuchu Xu proposed the research concept and design, drafted the initial manuscript, and conducted critical revisions on key academic content.

Xiaoxia Wang performed supplementary experiments in the later stages.

Xingya Yan completed experimental material preparation and executed data collection.

Xiaona Yin provided project funding and conducted experiments.

Xi Wang prepared experimental materials and conducted data collection.

Fangbin Du responsible for literature review.

Yinling Jiang performed statistical data analysis.

Xiaoqiong Wang assisted with statistical analysis, offered constructive feedback on the manuscript draft, and approved the final version for publication.

Yongsheng Wang Provided technical support for experimental procedures, participated in manuscript discussions, and approved the final manuscript.

## Funding

This study was supported by the Bengbu Medical University’s Natural Science Foundation (2023byzd248); the Scientific Research Program of Higher Education Institutions in Anhui Province (2024AH050703); Hefei Second People’s Hospital‐Level Topics (2024ykc001, 2024ykc002); Hefei Health and Wellness’s Applied Medical Research Program (Hwk2023zd012); and the Baiqun Public Welfare Fund (BFC‐QYWL‐QL‐20240906‐02). Grant Bengbu Medical University’s Natural Science Foundation (2023byzd248) and Grant Hefei Second People’s Hospital‐Level Topics (2024ykc001,2024ykc002) supported sample collection; Grant Scientific Research Program of Higher Education Institutions in Anhui Province (2024AH050703) funded experimental reagent purchase and result verification. Hefei Health and Wellness’s Applied Medical Research Program (Hwk2023zd012) supported sample collection and data sorting. Grant Baiqun Public Welfare Fund (BFC‐QYWL‐QL‐20240906‐02) funded experimental reagent purchase and result verification.

## Disclosure

All authors participated in the peer review process and approved the final version of the manuscript.

## Ethics Statement

This research was conducted in accordance with the Declaration of Helsinki and approved by the Ethics Committee of The Second People’s Hospital of Hefei (No. 2023—Scientific Research—048).

## Consent

The authors have nothing to report.

## Conflicts of Interest

The authors declare no conflicts of interest.

## Data Availability

The research piece offers the processing data essential for supporting the findings made. Upon reasonable request, data sets might be retrieved from the relevant authors.
